# The fast calibration model for dosimetry with an electronic portal imaging device

**DOI:** 10.1002/acm2.13599

**Published:** 2022-07-25

**Authors:** Stephanie Schade, Rita Engenhart‐Cabillic, Klemens Zink, Damian Czarnecki

**Affiliations:** ^1^ Department of Radiotherapy and Radiation Oncology University Medical Center Giessen and Marburg Klinikstrasse Giessen Germany; ^2^ Institute of Medical Physics and Radiation Protection University of Applied Sciences Giessen Wiesenstrasse Giessen Germany; ^3^ Department of Radiotherapy and Radiation Oncology University Medical Center Giessen and Marburg Klinikstrasse Giessen Germany; ^4^ Marburg Ion Therapy Center (MIT) Albrecht‐Kossel‐Strasse Marburg Germany

**Keywords:** calibration, EPID, in vivo dosimetry, transit dosimetry

## Abstract

**Purpose:**

The aim of this study was to develop an algorithm that corrects the image of an electronic portal imaging device (EPID) of a linear accelerator so that it can be used for dosimetric purposes, such as in vivo dosimetry or quality assurance for photon radiotherapy. For that purpose, the impact of the field size, phantom thickness, and the varying spectral photon distribution within the irradiation field on the EPID image was investigated.

**Methods:**

The EPID measurements were verified using reference measurements with ionization chambers. Therefore, absolute dose measurements with an ionization chamber and relative dose measurements with a detector array were performed. An EPID calibration and correction algorithm was developed to convert the EPID image to a dose distribution. The algorithm was validated by irradiating inhomogeneous phantoms using square fields as well as irregular IMRT fields.

**Results:**

It was possible to correct the influence of the field size, phantom thickness on the EPID signal as well as the homogenization of the image profile by several correction factors within 0.6%. A gamma index analysis (3%, 3 mm) of IMRT fields showed a pass rate of above 99%, when comparing to the planning system.

**Conclusion:**

The developed algorithm enables an online dose measurement with the EPID during the radiation treatment. The algorithm is characterized by a robust, non‐iterative, and thus real‐time capable procedure with little measuring effort and does not depend on system‐specific parameters. The EPID image is corrected by multiplying three independent correction factors. Therefore, it can easily be extent by further correction factors for other influencing variables, so it can be transferred to other linear accelerators and EPID configurations.

## 1 INTRODUCTION

National and international associations (AAPM, ICRU, DIN) recommend dose verification during irradiation while using modern irradiation techniques such as intensity modulated radiation therapy (IMRT) and volumetric modulated arc therapy (VMAT).^1^, ^2^, ^3^ The planned dose distribution can be validated according to the clinical procedure by using phantoms for dose validation before the treatment. It is also possible to measure the planned dose during irradiation selectively with thermo‐luminescence detectors (TLD). Neither method provides the ability to monitor patient dose during irradiation. Therefore, it would be practical to be able to use a measurement system during irradiation, that can measure and verify the patient plan during irradiation and take into account anatomical changes. For this reason, it would be important to use detectors that have a high spatial resolution, and which do not interfere with clinical operation during irradiation. Electronic portal imaging devices (EPID), which are installed practically in all modern linear accelerators, could fulfil the points already mentioned and could be used for dose verifications. The EPID image must be recalibrated by considering the detector response in a clinical radiation field and the preprocessing of the EPID image, like smoothing. Several publications have shown that dosimetric measurements are possible by recalibrating the EPID image.^4^, ^5^, ^6^, ^7^, ^8^, ^9^, ^10^

There are a set of parameters which effect the calibration that need to be investigated for calibration. Many of these have already been evaluated by other research groups.^11^, ^12^ Grein et al.^12^ showed different influences on the EPID image—effect of buildup, dose linearity, field size response, sampling of rapid multi‐leaf collimator (MLC) leaf speeds, response to dose‐rate fluctuations, memory effect, and reproducibility. Moreover, Grein^11^ investigated the influence of the field sizes and phantom thicknesses on the EPID image. Furthermore, other research groups have developed algorithms and models to correct the EPID image for dosimetric purposes. Most of these methods are based on an iterative approach.^9^, ^13^, ^14^, ^15^, ^16^, ^17^ This usually results in a good approximation but extends the computing time depending on the number of iterations. The correction model developed by Chang et al.^13^ which iteratively corrects the EPID image, uses a phantom scattering factor. This phantom scattering factor is based on the work of Keller et al.,^19^ Essers et al.,^20^ and Heijmen et al.^21^ who showed that scattering is strongly dependent on radiation field size and irradiated medium. Ghosting, beam inhomogeneities, energy spectrum, field size, and phantom thickness were investigated by Nijsten^14^ to correct the EPID image iteratively. The energy spectrum correction depends on off‐axis position and phantom thickness. The correction is accomplished by using transmission measurements in dependence of the irradiated patient volume and the irradiated field size. All correction factors are based on measurements. Transmission measurements were performed with polystyrene slabs of different thickness positioned at the isocenter. The profile correction is performed radial symmetrically to obtain a 2D portal dose correction. In the work of Alhazmi et al.^9^ pixel sensitivity is corrected by using a radial symmetric kernel based on a 1D Gaussian distribution as published by Greer et al.^15^ and Podesta et al.^22^ Field sizes and penumbra regions are also described and corrected by Podesta et al.^22^ by using two convolution kernels. In the publication of Greer et al.^15^ a radial symmetric map is used, which is based on a fourth‐degree polynomial to correct off‐axis deviation and pixel sensitivity. In the work published by Parent et al.^16^ three possible calibration models for the EPID image were investigated: flood field correction, Monte Carlo‐based correction and water slab calibration. Liebich et al.^18^ investigated the influences of irradiated dose, dose rate, field size, and reproducibility on the EPID image. A correction map at depth of maximum dose in polymethylmethacrylat (PMMA) is used to adjust the EPID image.

As it is already shown in previous studies, there are many different approaches to calibrate the EPID image for dosimetric purposes. An important aspect of EPID calibration, which is rarely discussed in the literature, is the impact of phantom thickness dependent scattering. Chang et al.^13^ determined a phantom correction factor, but not with reference to the scattering at different patient thicknesses. Nijsten et al.^14^ corrected the patient thickness only by correcting the beam profile. Therefore, since EPID has an energy‐dependent detector response, it is important to investigate the detector response as a function of the irradiated patient thickness. The irradiated patient changes the spectral distribution of the radiation field, due to beam hardening and attenuation.

The aim of this work was to investigate quantities which have a major effect on the EPID image in particular the beam hardening due to the patient thickness in order to develop an EPID calibration method for absolute dosimetry with high accuracy. For this reason, the EPID image was investigated as a function of field size and phantom thickness. Furthermore, the homogeneity of the beam profile as a function of different phantom thicknesses was investigated. The functionality and robustness of the developed calibration method was validated with IMRT‐fields and an inhomogeneous phantom.

## 2 MATERIAL AND METHODS

### 2.1 General formalism and definitions

To determine the absorbed energy dose in water $D_{w}^{f_{\mathrm{clin}},d_{\mathrm{clin}}}\left(x,y\right)$ of a clinical radiation field *f*_clin_ behind a patient of thickness *d*_clin_ from an acquired EPID image $I^{f_{\mathrm{clin}},d_{\mathrm{clin}}}\left(x,y\right)$, the following equation has been used:

(1) $$D_{w}^{f_{\mathrm{clin}}d_{\mathrm{clin}}}\;\left(x,y\right)=\;N\;\frac{I^{f_{\mathrm{clin}}d_{\mathrm{clin}}}\left(x,y\right)}{s_{\mathrm{clin}}}\;k_{f}\;k_{d}\;k_{\mathrm{profile}},$$

where *N* is the calibration factor for the conversion of the EPID image pixel values $I^{f_{\mathrm{ref}},d_{\mathrm{ref}}}\left(x\;=\;0,y\;=\;0\right)$ into absorbed dose to water $D_{w}^{f_{\mathrm{ref}},d_{\mathrm{ref}}}\left(x\;=\;0,y\;=\;0\right)$ at the center of the radiation field (x=0cm,y=0cm) under reference patient thickness *d*_ref_ and reference radiation field size *f*_ref_. Moreover, all images have to be divided by the pixel scaling factor *s*_clin_, which is used by the image processing software to convert the acquired EPID signal into a displayable image. $k_{f}$ is the field size correction factor, $k_{d}$ phantom correction factor and *k*_profile_ correction factor for inhomogeneity of the beam profile. The calibration factor *N* was calculated from the following equation:

(2) $$N\;=s_{\mathrm{ref}}\;\;\frac{D_{w}^{f_{\mathrm{ref}},d_{\mathrm{ref}}}\left(x\;=\;0,\;y\;=\;0\right)}{I^{f_{\mathrm{ref}},d_{\mathrm{ref}}}\left(x\;=\;0,y\;=\;0\right)},$$

where $I^{f_{\mathrm{clin}},d_{\mathrm{clin}}}\left(x\;=\;0,y\;=\;0\right)$ is the pixel value at the center of the radiation field under reference conditions and *s*_ref_ is the pixel scaling factor for the reference field. In this work, we chose $f_{\mathrm{ref}}=\;10\;\times \;10\;cm^{2}$ and $d_{\mathrm{ref}}=\;0\;\mathrm{cm}$ for the reference conditions, that is, no phantom present in the radiation field.

The size of the clinically applied radiation field *f*_clin_ is estimated from the image $I^{f_{\mathrm{clin}},d_{\mathrm{clin}}}\left(x,y\right)$ using the OTSU method^23^ and assigned to a square field that encloses the entire field. In the following section, the correction factors $k_{f}$, $k_{d},$ and *k*_profile_ are defined.

### 2.2 Correction factor for field size $k_{f}$

The correction factor for field size $k_{f}$ is intended to correct the over‐ and under‐response of the EPID image for different field sizes *f*_clin_. $k_{f}$ is defined in the following equation:

(3) $$k_{f}=\frac{D_{w}^{f_{\mathrm{clin}},d_{\mathrm{ref}}}\left(x=0,y=0\right)/{\bar{I}}^{f_{\mathrm{clin}},d_{\mathrm{ref}}}}{D_{w}^{f_{\mathrm{ref}},d_{\mathrm{ref}}}\left(x=0,y=0\right)/{\bar{I}}^{f_{\mathrm{ref}},d_{\mathrm{ref}}}}.$$

The values ${\bar{I}}^{f_{\mathrm{clin}},d_{\mathrm{ref}}}$ and ${\bar{I}}^{f_{\mathrm{ref}},d_{\mathrm{ref}}}$ were calculated by averaging the individual gray values in a small area (1 cm^2^ × 1 cm^2^) at the beam central axis of the EPID image. For field sizes *f*_clin_ for which no correction factor $k_{f}$ was measured, the data were linear interpolated.

### 2.3 Correction factor for phantom thickness $k_{d}$

The phantom thickness correction factor $k_{d}$ is intended to correct the over‐ and under‐response of the EPID due to different phantom thicknesses *d*_clin_ irradiated by the linear accelerator.

(4) $$k_{d}=\frac{D_{w}^{f_{\mathrm{clin}},d_{\mathrm{clin}}}\left(x=0,y=0\right)/{\bar{I}}^{f_{\mathrm{clin}},d_{\mathrm{clin}}}}{D_{w}^{f_{\mathrm{clin}},d_{\mathrm{ref}}}\left(x=0,y=0\right)/{\bar{I}}^{f_{\mathrm{clin}},d_{\mathrm{ref}}}}.$$

The correction factor is calculated for various clinical field sizes *f*_clin_ and phantom thicknesses *d*_clin_. For *f*_clin_ and *d*_clin_ for which no correction factor $k_{d}$ was measured, the corresponding value was interpolated from the measured data with a third‐degree polynomial. *d*_clin_ must be adjusted for densities that do not correspond to that of water. The phantom thickness *d*_clin_ was adapted to the density of the material in relation to the density of RW3.

### 2.4 Correction factor for inhomogeneities in the beam profile $k_{\mathrm{profile}}$

The image processing software of the EPID flattens the acquired image data. The software takes an image every 320 ms and averages the images taken and saves this as one image. This image is additionally corrected with a dark image, which is created by the program before each recording. This serves as an offset correction.^17^ Thus, the profiles of an image do not correspond to the dose distribution of the radiation field. The correction factor *k*_profile_ should correct the profile of the image according to the lateral dose distribution. The correction factor depends on the irradiated patient thickness *d*_clin_ and is defined in the following equation:

(5) $$k_{\mathrm{profile}}\;\left(x,y,d_{\mathrm{clin}}\right)=\frac{D_{\mathrm{rel}}^{f_{\max},d_{\mathrm{clin}}}\left(x,y\right)}{I^{f_{\max},d_{\mathrm{clin}}}\left(x,y\right)}.$$

$D_{\mathrm{rel}}^{f_{\max},d_{\mathrm{clin}}}\left(x,y\right)$ is measured with a maximum field size *f*_max_. For field sizes not corresponding to *f*_max_, *k*_profile_ is scaled up or down to the required field size *f*_clin_. For smaller or larger field sizes, the dataset of *k*_profile_ is compressed or expand laterally, respectively. This approximation was chosen to keep the measurement effort during calibration as low as reasonably necessary. To reduce uncertainties in lateral measurements, *k*_profile_ values could be introduced for different field sizes, for example, large, medium, and small field sizes.

## 3 MATERIAL

All measurements were performed using an Elekta Synergy (Elekta Oncology Systems, Crawley, UK) linear accelerator (linac) with a 120‐leaf MLC using 6 MV photon beams. For this work, all measurements were done with the highest dose rate of 500 MU/min. The linac was equipped with an Elekta *i*ViewGT™ a‐Si EPID. The EPID has a fixed distance to the source (SDD = 160 cm) and an active imaging area of 41 × 41 cm^2^ consisting of 1024 × 1024 detector elements, resulting in a pixel resolution of 0.4 mm. The image acquisition software was *i*ViewGT™ software (version 3.4, Elekta). The EPID detector takes 2.3 images every second, the software averages these images, so‐called subframes, to one image after irradiation. The EPID was irradiated with a protective cover. As described by Parent et al.,^16^ the protective cover has no significant influence on the measurement. All measurements were performed with a gantry angle of 0° to minimize the effects of gravity on the EPID detector. This also minimized the positioning inaccuracy of the EPID detector.

The calibration factor *N* and the correction factors $k_{f}$, $k_{d},$ and *k*_profile_ were determined experimentally using the reference conditions presented in Table 1. The field sizes mentioned in this work are nominal field sizes whose size is defined at the isocenter with 100 cm distance to the source. All dose measurements and calculations were carried out at a distance of 160 cm.

**TABLE 1:** Measuring set‐up to determine the calibration factor *N* and the correction factors $k_{f}$

Determined quantity	Field size *f*_clin_	Phantom thickness *d*_clin_
*N*	10 × 10 cm^2^	0 cm
$k_{f}$	4 × 4 – 26 × 26 cm^2^	0 cm
$k_{d}$	5 × 5 –20 × 20 cm	0–20 cm
*k*_profile_	*f*_max_ = 15 × 15 cm^2^	0–20 cm

*Note*: $k_{d}$ and *k*_profile_ with a fixed distance between radiation source and detector/EPID of 160 cm.

All dose values $D_{w}^{f_{\mathrm{clin}},d_{\mathrm{clin}}}\left(x\;=\;0,y\;=\;0\right)$ at the center of the radiation field were measured with an SSD = 160 cm using the ionization chamber T31013 (PTW, Freiburg, Germany). For these measurements, a build‐up layer of 1.6 cm RW3 (PTW, Freiburg) was used to measure at the depth dose maximum.

$k_{f}$ values were measured for field sizes from 4 × 4 cm^2^ to 26 × 26 cm^2^ by increasing the field length in the *x* and *y* direction by 2 cm.

The correction factor $k_{d}$ was determined for different phantom thicknesses *d*_clin_ and field sizes. $k_{d}$ values were measured for phantom thicknesses from 0 to 20 cm with a step size of 4 cm using RW3 plates (30 × 30 × 1 cm^3^) for different radiation fields (5 × 5 cm^2^, 10 × 10 cm^2^, 15 × 15 cm^2^, and 20 × 20 cm^2^).

A 2D ionization chamber array (Octavius detector 1500, PTW) was used to measure beam profiles for the correction factor *k*_profile_. The maximum possible field size *f*_max_ which could be measured with this detector array at a distance of 160 cm from the radiation source was 15 × 15 cm^2^ at the isocenter. The correction factor *k*_profile_ was determined for irradiated phantom thicknesses of 0 cm up to 20 cm with a step size of 4 cm.

### 3.1 Validation measurements

The EPID calibration algorithm was validated under clinical irradiation conditions. First, the dose calculation of the algorithm was validated with a three density phantom. It consists of three different densities, which are similar to the density of tissue, fat, and bone.

A nine‐field IMRT was studied to investigate the performance of the dose calculation algorithm in a radiation field consisting of several irregular fields. It was a clinical patient plan of a mediastinum with simultaneously integrated boost and a collimator angle of 5°. No patient or phantom was in the beam path during the measurement. The measurements with irregular field geometries were compared with the dose distribution of the treatment planning system (TPS) using the gamma index (3, 3 mm), applying the software Verisoft (version 7.1, PTW).^17^ The dose distribution from the planning system Pinnacle^3^ (version 16.2, Philips, Amsterdam, the Netherlands) was compared with the corrected dose distribution from the EPID image. The dose distribution of the EPID was obtained by replicating the system in the planning software and calculating the dose at the measurement plane (SSD = 160 cm).

## 4 RESULTS

### 4.1 Correction factor for field size $k_{f}$

**FIGURE 1:**
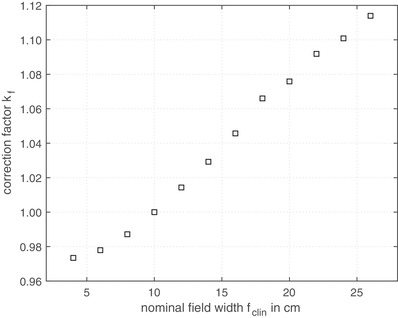
Measured correction factor for field size $k_{f}$ according to Equation (3) as a function of the nominal field size defined at the isocenter

Figure 1 shows the correction factor $k_{f}$ with the reference field size $f_{\mathrm{ref}}=\;10\;\times \;10\;cm^{2}$ for different quadratic field sizes *f*_clin_. As can be seen in Figure 1, the EPID has an over response in small radiation fields and an under response in radiation fields larger than 10 × 10 cm^2^.

### 4.2 Correction factor for phantom thickness $k_{d}$

**FIGURE 2:**
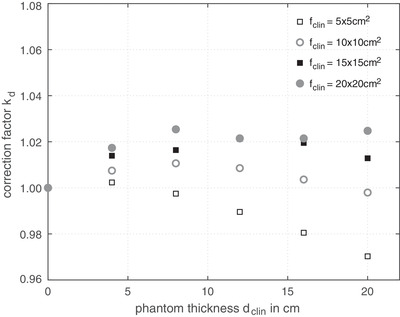
Measured correction factor $k_{d}$ according to Equation (4) with a reference phantom thickness $d_{\mathrm{ref}}=\;0\;cm$. $k_{d}$ values are shown as a function of the irradiated phantom thickness for four different field sizes *f*_clin_

Figure 2 shows the measured correction factor $k_{d}$ with the reference phantom thickness $d_{\mathrm{ref}}=\;0\;\mathrm{cm}$ as a function of irradiated phantom thickness *d*_clin_. As can be seen the values of the correction factor $k_{d}$ strongly depend on the irradiated field size. It should be noted that for the smaller radiation field 5 × 5 cm^2^, the over‐response of the EPID increases with increasing phantom thickness *d*_clin_. For large field sizes (> 10 × 10 cm^2^), the values for $k_{d}$ are between 1.1 and 1.25.

### 4.3 Correction factor for inhomogeneities in the beam profile $k_{\mathrm{profile}}$

**FIGURE 3:**
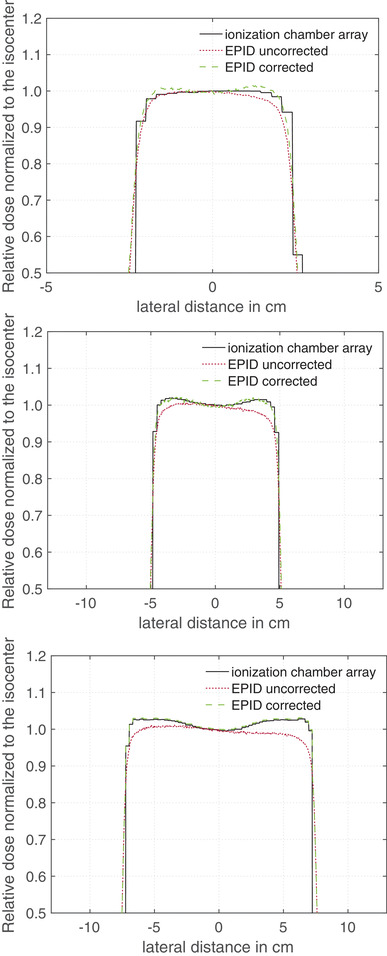
Lateral dose profiles normalized to the dose at the center of the radiation field measured with a detector array and electronic portal imaging device (EPID) for three different field sizes: (a) 5 × 5 cm^2^, (b) 10 × 10 cm^2^, and (c) 15 × 15 cm^2^. The black line and the red dotted line represent the profile measurements with the ionization chamber array and EPID, respectively. The green dashed line shows the EPID measured curve corrected with the correction factor *k*_profile_

Figure 3a–c provides lateral dose profiles measured with a detector array (black line) and EPID (red dotted line) normalized to the dose at the center of the radiation field. The green dashed line in Figure 3 presents the EPID profile corrected by the correction factor *k*_profile_ from Equation (5).

**FIGURE 4:**
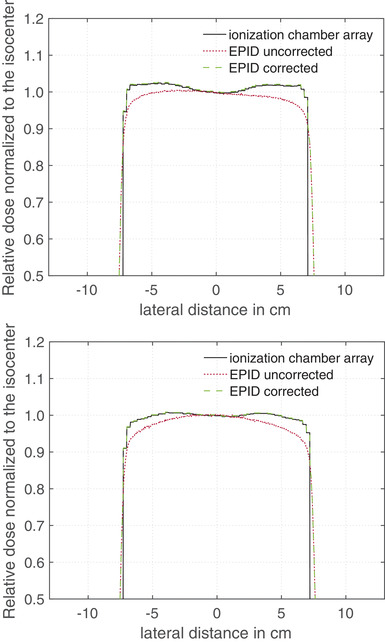
Lateral dose profiles normalized to the dose at the center of the radiation field measured with a detector array and electronic portal imaging device (EPID) for a 15 × 15 cm^2^ radiation field. The black line and the red dotted line represent the profile measurements with the ionization chamber array and EPID, respectively. The green dashed line shows the EPID measured curve corrected with the correction factor *k*_profile_. In (a) measurements 4 cm RW3 and in (b) measurements 20 cm RW3 were placed in the radiation field

Figure 4 shows the measured relative dose profiles in the same way as Figure 3, but in this case 4 cm RW3 (4(a)) and 20 cm RW3 (4(b)) were placed at the isocenter between the radiation source and the detector. Comparing the relative dose profiles of Figure 4a,b, it can be seen that the dose profile correction depends strongly on the irradiated phantom thickness *d*_clin_.

As can see from Figures 3 and 4, the uncorrected EPID profile (red dotted line) shows a large deviation from the profile measured with the detector array—over all investigate field sizes (from 5 × 5 up to 17 × 17 cm^2^) and phantom thickness (from 0 up to 20 cm) a maximum deviation of 12% was observed. The largest deviation between the relative dose profile from EPID and detector array measurements were observed at the field edge. When the correction factor *k*_profile_ was applied to the EPID data, the deviation at the field edge could be reduced to a maximum of 0.3% for field sizes of 10 × 10 cm^2^ or higher. For smaller field sizes, the deviations are larger, as can be seen in Figure 3a. For the smallest measured field size, the maximum deviation was 1.6%.

### 4.4 Validation of the dose calibration algorithm

**FIGURE 5:**
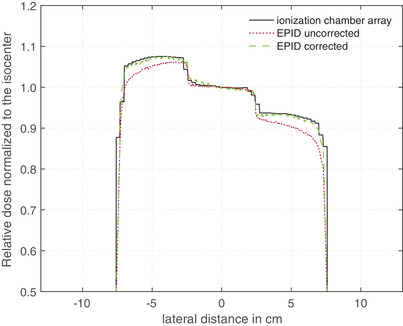
Lateral dose profiles normalized to the dose at the center of the radiation field measured with a detector array and electronic portal imaging device (EPID) for a 15 × 15 cm^2^ radiation field. It was measured behind a phantom with three inhomogeneities (air, tissue, bone‐like materials) with a detector array (black line) and EPID (red dotted line). The green dashed line shows the EPID measured curve corrected with the correction factor *k*_profile_

A phantom with three inhomogeneities (air, tissue, and bone‐like materials) was irradiated by a 15 × 15 cm^2^ radiation field, to validate the dose profile corrections for inhomogeneous phantoms. Figure 5 presents the relative dose profiles measured behind the phantom with the ionization chamber array (black line) and EPID (red dotted line). The corrected EPID dose profile is represented by the green dashed line. Uncorrected EPID profiles deviate from ionization chamber measurements in mean about 5% (maximum deviation 20%) − corrected values in mean about 0.6% (maximum deviation 3%).

**FIGURE 6:**
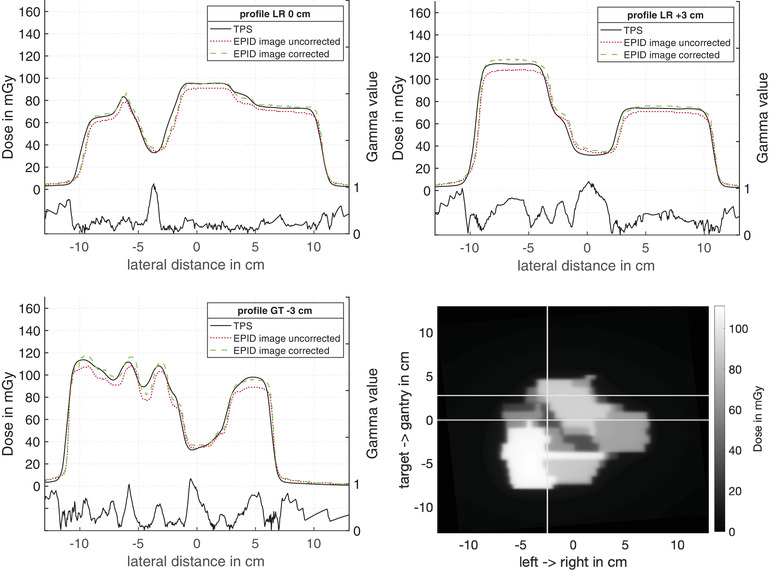
Intensity modulated radiation therapy (IMRT) field dose distribution measured with electronic portal imaging device (EPID). The lateral dose distribution of a clinical IMRT field was calculated according to Equation (1). (a)–(c) show the dose profile of the uncorrected (dotted lines), corrected EPID image (dashed lines), and TPS signal (solid lines) in different directions. In addition, the result of the gamma analysis was shown below the profile. The uncorrected dose profiles were calculated by setting the correction factors of Equation (1) to 1. The white line in (d) marks the line from which the profiles in (a), (b) and (c) were created. (a) in left–right direction in isocenter. (b) in the left–right direction with 3 cm shift in the gantry direction. (c) in gantry target orientation with a 3 cm shift in the left direction. (d) show the corrected dose distribution over the entire radiation field

**FIGURE 7:**
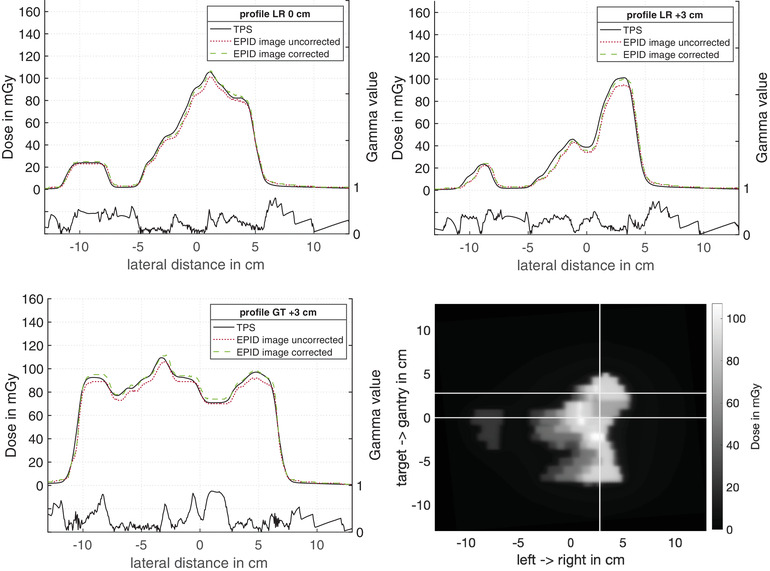
Intensity modulated radiation therapy (IMRT) field dose distribution measured with electronic portal imaging device (EPID). The lateral dose distribution of a clinical IMRT field was calculated according to Equation (1). (a)–(c) show the dose profile of the uncorrected (dotted lines), corrected EPID image (dashed lines) and treatment planning system (TPS) signal (solid lines) in different directions. In addition, the result of the gamma analysis was shown below the profile. The uncorrected dose profiles were calculated by setting the correction factors of Equation (1) to 1. The white line in (d) marks the line from which the profiles in (a)–(c) were created. (a) In the left–right direction in isocenter. (b) In the left–right direction with 3 cm shift in the gantry direction. (c) In gantry target orientation with a 3 cm shift in the right direction. (d) shows the corrected dose distribution over the entire radiation field

Moreover, the algorithm was validated using a clinical IMRT plan of a mediastinum with simultaneously integrated boost. In Figures 6 and 7, two EPID images of IMRT fields are presented. The white lines in Figures 6d and 7d mark the line from which the profiles in Figures 6 and 7a–c were created. Part (a) shows a profile in the left–right direction through the center of the radiation field, Part (b) shows a profile in the left–right direction with a shift of 3 cm in the target direction for Figure 6 and a shift of 3 cm in the gantry direction for Figure 7. Part (c) shows the profile in the gantry target direction with a displacement of 3 cm in the left direction for Figure 6 and in the right direction for Figure 7. Figures 6 and 7a–c show dose profiles calculated from the EPID image and the reference dose distribution from the planning system. The dotted lines are dose profiles calculated by setting the correction factors of Equation (1) to 1. The dashed lines in Figures 6 and 7a–c show the dose profiles corrected by the correction factors $k_{f}$, $k_{d},$ and *k*_profile_. The solid lines show the dose profiles from the TPS. The corrected EPID image dose distributions were compared to the dose distributions from the planning system with the gamma criterium (3%, 3 mm). The distribution of the gamma values for the respective profile can be found in the corresponding figures below the profiles. Only a few points within the profiles show a deviation of more than 1. Gamma analysis of the investigated IMRT fields measured by EPID with a Gamma criterion of (3%, 3 mm) and (2%, 2 mm) resulted in a pass rate of 99% and 94%, respectively.

## 5 DISCUSSION

In this study, an algorithm for EPID image correction was developed with a non‐iterative correction model consisting of three correction factors. The individual factors which influencing the EPID image could be investigated separately with the calibration formalism presented in this work.

As can been seen in Figure 1, the EPID response is strongly dependent on the field size *f*_clin_. These findings are in good agreement with previous studies^14^, ^18^ which predicting an EPID over response for small field sizes and an under response for large field sizes.

In this work, it could be shown that the individual influencing variables such as field size *f*_clin_ and patient thickness *d*_clin_ have a mutual influence on the EPID response as well as on the image processing software. As can be seen in Figure 2 a phantom thickness of 4 cm led to an under response of the EPID for all investigated field sizes *f*_clin_. However, a much thicker phantom $d_{\mathrm{clin}}=\;20\;\mathrm{cm}$ resulted in a 3% over response for the small field size 5 × 5 cm^2^. As already mentioned by Liebich et al.,^18^ a large impact of the irradiated patient thickness *d*_clin_ on the detector response was observed. Nijsten et al.^14^ was able to show that there is an under‐response of the EPID with increasing phantom thickness and off‐axis position.

The influence of the EPID image smoothing has already been discussed in other publications.^18^ It has been found that a correction is necessary because Elekta pre‐processing algorithm smoothed the EPID signal. This minimizes the inhomogeneities of the acquired EPID image. As can be seen in Figure 4, the red dotted line is more homogeneous and the signal enhancements on the outer sides (black line) are smoothed away, even slightly inverted. In this study, it could be observed that the image smoothing strongly correlates with the irradiated phantom thickness, because the inhomogeneity of the dose profile changes with the irradiated thickness (see Figure 4). Therefore, the correction factor *k*_profile_ had to be determined depending on the phantom thickness. The results indicate that the irradiated phantom thickness *d*_clin_ is a very important factor when transforming the EPID signal into a dose distribution and should be taken into consideration in all EPID calibration algorithms for relative as well as absolute dose calculations. In Figure 3, it can be seen that resizing the correction factor *k*_profile_ to the field sizes of 10 × 10 cm^2^ results in a maximum deviation of 0.3% from measurements with the reference detector array. A larger deviation was observed for smaller field sizes, for example, for a field size of 5 × 5 cm^2^ a maximum deviation of 1.6% was observed at the radiation field edge. This algorithm can be extent by further correction factors *k*_profile_ calculated from smaller field sizes. This would prevent the resizing of the correction factor $k_{\mathrm{profile}}\;$to such large extent and thus reduce the deviation of the EPID signal from ionization chamber measurements.

Furthermore, the study showed that the dose calibration algorithm was applicable to irregular IMRT fields (Figure 6). The measured dose distribution showed a pass rate of 99% and 94% with a Gamma index of 3% and 3 mm and 2% and 2 mm, respectively. Thus, the algorithm developed in this work has a pass rate comparable to that accomplished in the studies by Nijsten et al.,^14^ Greer et al.,^15^ and Deshpande et al.^10^

Similar to the work by Nijsten et al.,^14^ this algorithm takes into account the impact of the field size as well as the patient thickness on the EPID signal and perform a lateral profile correction. However, the EPID calibration of Nijsten et al.^14^ follows a different approach. The image is corrected not only by multiplying correction factors, but also by convolution kernel and an iterative deconvolution of the EPID image. Several studies^23^, ^24^ have modeled the field size‐dependent response of EPID detectors by convolution kernels similar to the approach by Nijsten et al.^14^ On the other hand, the aim of this work was to develop a non‐iterative calibration method without a convolution approach. Just like the algorithm published by Nijsten et al.,^14^ this method should only be based on measurements. Besides that, the measurement effort should be as low as possible, and the algorithm should retain its simplicity and good feasibility in order to be well integrated into the clinical routine.

Therefore, ghosting, dose rate variation, and memory effect were not taken into account in this algorithm. No significant impact on the dose calculation were observed from these effects. However, the impact of ghosting, dose rate variations, and memory effect were studied in previous studies in detail.^14^, ^18^

One should note that the IMRT measurements were performed without a patient or phantom in the radiation field. Thus, $k_{d}$ = 1 and the *k*_profile_ correction factor was selected for d= 0 cm. In case of a patient in the radiation field, the correction factors $k_{d}$ and *k*_profile_ should be adjusted according to the patient thickness *d* along the radiation field. The patient thickness can be calculated from the CT data of the patient. Due to the inhomogeneity of the patient, the correction factor would be depending on the position x,y of the radiation field like in the measurements with the phantom with three inhomogeneities presented in Figure 5.

Moreover, the largest possible field size was not chosen as the reference field of the calibration method, but the field size 10 × 10 cm^2^, which is used also in all known dosimetry protocols as the reference field size.

## 6 CONCLUSION

The algorithm developed in this work performs a cross‐calibration of the EPID images against ionization chamber measurements. The algorithm consists of a calibration factor measurement under reference conditions and several correction factors for non‐reference conditions. This formalism is closely related to the formalism developed by Alfonso *et al*.^25^ for the reference dosimetry of small and non‐standard fields.

Using this formalism, it was possible to separate different influencing parameters on the EPID signal. The study was able to show how the field sizes and irradiated phantom thicknesses affects the EPID response. The algorithm was validated in addition to simple fields with clinically more relevant IMRT fields. The gamma analysis of all nine test fields showed a pass rate of more than 99% with a gamma index of 3% and 3 mm.

A major advantage of this algorithm is its independence from the linear accelerator system. This algorithm can be applied to all linac and EPID configurations, no further conditions have to be considered. With a small measurement effort, the EPID can be calibrated to match ionization chamber measurements. This algorithm can be easily integrated into the clinical routine, due to the small number of calibration measurements with ionization chambers and detector arrays. It offers the possibility to use it for quality assurance measurements in clinical routine. The algorithm is characterized by a non‐iterative procedure, which is real‐time capable due to the short computing time. Therefore, it has the potential to be used as an online dosimeter during radiation treatment, which is not in the beam path between radiation source and patient. Furthermore, it is interesting to compare the algorithm presented in this work with deconvolution‐based algorithms to investigate the limitations and advantages of an EPID calibration which is based on correction factors. In addition, the long‐term stability should be checked by further measurements.

## CONFLICT OF INTEREST

The authors declare that there is no conflict of interest that could be perceived as prejudicing the impartiality of the research reported.

## AUTHOR CONTRIBUTIONS

Stephanie Schade performed the software development and measurements and was responsible for writing and creating the original design. Damian Czarnecki collaborated in software development and data analysis and contributed to the original draft. All authors participated in revising the manuscript and approved the final manuscript.
